# Factors Related to Antibiotic Supply without a Prescription for Common Infections: A Cross-Sectional National Survey in Sri Lanka

**DOI:** 10.3390/antibiotics10060647

**Published:** 2021-05-28

**Authors:** Shukry Zawahir, Sarath Lekamwasam, Parisa Aslani

**Affiliations:** 1 Central Clinical School, Faculty of Medicine and Health, The University of Sydney, Sydney, NSW 2006, Australia; 2 The University of Sydney School of Pharmacy, Sydney, NSW 2006, Australia; parisa.aslani@sydney.edu.au; 3 Population Health Research Centre, Department of Medicine, Faculty of Medicine, University of Ruhuna, Galle 80000, Sri Lanka; slekamwasam@gmail.com

**Keywords:** pharmacy, pharmacist, Sri Lanka, antibiotics, dispensing, common infections, attitudes

## Abstract

Inappropriate antibiotic use is a problem in Sri Lanka. We investigated pharmacy staff’s attitudes towards antibiotic supply for common infections in Sri Lanka. A self-reported cross-sectional survey was conducted among a random sample (*n* = 369) of pharmacies. We assessed staff’s beliefs and attitudes to antibiotic supplying for common infections (common cold and cough, sore throat, diarrhoea, wound and urinary tract infections (UTI)). Pharmacists (*n* = 210; 79%) and pharmacy assistants (*n* = 55: 21%) responded. About 30% (80/265) had supplied antibiotics without a prescription for common infections, including common cold (15.8%), sore throat (13.6%) and diarrhoea (10.2%). Overall, pharmacists were less likely to supply than non-pharmacists. Pharmacy staff with more positive beliefs about their professional competency to supply and monitor antibiotic use were more likely to supply antibiotics without a prescription for common cold (Adj.OR = 1.08; 95% CI: 1.01–1.15; *p* = 0.032), wound infections (Adj. OR = 1.06; 95% CI: 1.00–1.13; *p* = 0.059), and UTI (Adj.OR = 1.07; 95% CI: 0.99–1.15; *p* = 0.097). Pharmacy staff who believed in the effectiveness of antibiotics against common infections were more likely to supply antibiotics for common infections. Supply of antibiotics without a prescription was associated with staff’s beliefs about antibiotics’ effectiveness and their professional competency. Our findings could be used to strengthen regulatory strategies to improve practice.

## 1. Introduction

Globally, antibiotic consumption has increased by 35% between 2000 and 2010 [1]. The rate of antibiotic consumption correlates closely with the emergence of antibiotic resistance (ABR), in both hospital and outpatient settings [2,3]. Inappropriate antibiotic use is a major driver of ABR, increasing morbidity, mortality, and unnecessary healthcare costs [4,5], and representing one of the most important threats to global public health and patient safety [6,7]. This has led the WHO to develop a global action plan which includes a range of activities to raise awareness of the importance of protecting the efficacy of antibiotics and reducing inappropriate antibiotic use [8].

In the majority of countries worldwide, there is a legal requirement for antibiotics to be supplied only on prescription from physicians. However, in many countries, antibiotics can be purchased from community pharmacies without a prescription [9]. Inappropriate and illegal antibiotic supply for self-medication is a common practice in many low and middle-income countries (LMICs) [9,10]. Self-medication with antibiotics is closely associated with inappropriate antibiotic use [11]. In a recent survey of the Sri Lankan general public, nearly all participants (98%, 974/998) reported that they had used an antibiotic in the past and half of them (50%) claimed to have taken an antibiotic at least once in the three months prior to the survey [12]. Despite the fact that antibiotics cannot treat infections caused by viruses such as common cold, acute sore throat, runny nose and flu [13,14,15,16], the majority of respondents (76%) in the Sri Lankan study had used antibiotics inappropriately for these minor infections [12].

Easy access to antibiotics without a prescription has been identified as an important contributing factor to self-medication with these medicines [17]. Antibiotics are easily accessible from Sri Lankan community pharmacies without a prescription for common infections [18,19]. Studies have revealed several pharmacist-related factors associated with antibiotic supply without a prescription, including pharmacists’ lack of clinical training [20,21,22,23], and their beliefs that ABR is caused by factors other than supply of antibiotics from pharmacies [24,25,26,27], whilst pharmacists’ beliefs about the lack of benefits of antibiotics for upper respiratory infections (URTIs) have been associated with a decreased intention to supply antibiotics [25]. No studies have been done, however, to investigate the determinants of antibiotic supply without a prescription in Sri Lanka. Furthermore, the current knowledge generated elsewhere cannot be extrapolated to Sri Lanka due to the differences between community pharmacies in Sri Lanka and other parts of the world. As shown in a recent parallel study in Sri Lanka, half of the registered pharmacies were run by non-pharmacists [19], and most of the community pharmacists had gained an efficiency pharmacy training (apprentice pharmacist) under a senior pharmacist’s supervision [28]. Apprentice pharmacists do not have a tertiary qualification and do not complete a clinical training component. Only about 2% of the community pharmacists in Sri Lanka are pharmacy graduates [28]. This study aimed to evaluate pharmacy staff’s attitudes towards antibiotic supply without a prescription for common infections and investigate the association between attitudes and supply of antibiotics.

## 2. Results

### 2.1. Respondent Characteristics 

In total, 267 pharmacy staff (pharmacists and pharmacy assistants) completed the survey (response rate = 72%). Two incomplete questionnaires were excluded and 265 responses were included in the final analyses. Fifty-five (21%) respondents were categorised as pharmacy assistants (had no formal pharmacy qualification or training) and 210 (79%) as pharmacists. No significant difference was observed between pharmacy assistants and pharmacists in terms of personal and professional characteristics assessed except for the years of working experience. Pharmacists had worked for a longer period than assistants (χ^2^ (3, *n* = 265) = 26.85, *p* < 0.001). Among the pharmacists (*n* = 210), the majority had an efficiency (apprentice) pharmacy training (*n* = 140, 67%) and about 2% (*n* = 5) had a formal university degree in pharmacy. Most of the demographic characteristics of the participants (Table 1) have been previously reported [28].

### 2.2. Descriptive Statistics and Exploratory Factor Analysis

Table 2 shows the factor analysis data. All 43 attitudinal items were included in the initial exploratory factor analysis (EFA). Eleven items were removed (one at a time) from the final factor solution, if they did not correspond to other items in the factor or had a factor loading of ≤0.30 [29]. The EFA revealed five factors with the eigenvalues above 1, explaining 62.7% of the total variance in the observed variables. The final five-factor solution consisted of 32 attitude items and was consistent with the conceptual framework. 

Factor 1, Positive professional competency to supply and monitor, explained the pharmacy staff’s beliefs about their professional competency (e.g., due to their pharmacy training, knowledge, patient counselling ability and experiences) to supply antibiotics without a prescription and monitor people’s use of antibiotics (e.g., monitor side effects) without a prescription. Factor 2, Shared responsibility, focused on pharmacy staff’s beliefs that promoting appropriate use of antibiotics is a shared responsibility of various stakeholders, including policy makers, patients, pharmacists and doctors. Factor 3, Beliefs about effectiveness of antibiotics, explained participants’ beliefs about the effectiveness of antibiotics against a range of common conditions presented by people to community pharmacy staff, such as wound infections, common cold and cough, diarrhoea, acute sore throat, fever and UTI. 

Factor 4, Access and availability, centred on pharmacy staff’s opinions about the ability of people to easily access and receive antibiotics without a prescription from pharmacies to meet their needs if they do not have enough money or time to see a doctor. Contributing to these beliefs were the pharmacy staff’s beliefs that antibiotics can cure all bacterial infections, ABR is not a problem, and they were sufficiently trained on antibiotics in their pharmacy training. Factor 5, Appropriate and legal supply and use, explained participants’ attitudes towards antibiotics supply control strategies, and included beliefs that antibiotic supply without a prescription should be controlled by authorities, and that patients’ demands and misuse of antibiotics and pharmacists’ contribution towards appropriate use of antibiotics should be stopped (Table 2). 

All items loaded onto the expected factors with factor loadings greater than 0.4, except two items which loaded onto factor 4 (0.35 and 0.31) and one item onto factor 5 (0.37) with lower factor loadings. Weighted factor-based scales were calculated for all five factors (Table 3). The mid-point scale for each factor was calculated and it was used to compare the agreement or disagreement with the factor. Factor-based scales above the midpoint indicate agreement and vice versa. All weighted factor-based scales were slightly skewed (Table 3). 

The Cronbach’s alpha for Factor 1 was 0.95, indicating a very high level of internal consistency. The median score for each of the 10 items measuring beliefs in professional competency to supply antibiotics without a prescription and monitor antibiotics use (Factor 1) was 2 (IQR 1, 2) (Table 2), indicating that overall, the respondents disagreed with the statements measuring their beliefs in their professional competence to supply antibiotics without a prescription and monitor antibiotics use. The data also indicated that the median weighted factor-based scale for Factor 1 was below the mid-point value. This demonstrated that the respondents did not believe that they had the professional competency to supply antibiotics without prescription and monitor antibiotics use (Table 3). 

The Cronbach’s alpha for Factor 2 was 0.83, indicating good internal consistency. Pharmacy staff agreed with all four items (median 4 or 5) regarding responsibility for appropriate use of antibiotics being shared by various stakeholders, including health professionals, policy makers and consumers (Factor 2). The data further indicated that the median weighted factor-based scale for Factor 2 was above the mid-point value. This demonstrated that the majority of respondents believed that promoting appropriate use of antibiotics is a shared responsibility of all stake holders (Table 2 and Table 3). 

The Cronbach’s alpha for Factor 3 was 0.92, indicating high internal consistency. Overall, the respondents disagreed (median 1 or 2) with all of the six items measuring beliefs about effectiveness of antibiotics for different conditions (Factor 3) evaluated (wound infections, the common cold and cough, diarrhoea, acute sore throat, fever and UTI). The data also showed that the median weighted factor-based scale for Factor 3 was below the mid-point value. This demonstrated that the majority of the respondents did not believe in the effectiveness of antibiotics for the evaluated conditions (Table 2 and Table 3). 

The Cronbach’s alpha for Factor 4 was 0.76, indicating acceptable internal consistency. The median score for each of the seven items which measured access and availability was 1 or 2 (Factor 4), indicating disagreement of the respondents with supply of antibiotics to the public without a prescription. However, the median weighted factor-based scale for Factor 4 was above the mid-point value. This demonstrated that the majority of the respondents supported people’s ability to easily access and receive antibiotics from pharmacies without a prescription (Table 2 and Table 3). 

The Cronbach’s alpha for Factor 5 was 0.70, indicating acceptable internal consistency. The median score for each of the five items in Factor 5 was 4 or 5, indicating respondents’ overall high levels of agreement for the items measuring the appropriate and legal supply and use of antibiotics. The median weighted factor-based scale for Factor 5 was above the mid-point value, indicating that the majority of the respondents did support the appropriate and legal supply and use strategies (Table 2 and Table 3). 

There were no significant differences found in the respondents’ attitudinal factor scores between the three different groups of respondents, i.e., the pharmacists with proficiency qualification, the pharmacists with efficiency qualification, and non-pharmacists (Table S1). 

### 2.3. Self-Reported Antibiotic Supply without a Prescription

About 30% (80/265) of respondents reported that they had dispensed antibiotics without a prescription for the common infections, such as acute sore throat, common cold and cough, diarrhoea, wound infection and UTI, in the week prior to completing the survey. About 40% of the antibiotic supply without a prescription was for potentially viral infections, including common cold and cough (15.8%), acute sore throat (13.6%) and diarrhoea (10.2%). The reported antibiotic supply without a prescription for wound infection and UTI were 21.1% and 8.7%, respectively (Table 4). No significant differences were observed in antibiotic supply without a prescription between pharmacists with efficiency qualification and those with proficiency qualification.

### 2.4. Factors Related to Antibiotic Supply without a Prescription for Common Infections 

The multiple logistic regression results are shown in Table 4. Pharmacy staff with more positive beliefs about their professional competency to supply antibiotics without a prescription and monitor people’s antibiotic use were more likely to supply antibiotics without a prescription for common cold and cough (Adj.OR = 1.08; 95% CI: 1.01–1.15; *p* = 0.032), wound infections (Adj. OR = 1.06; 95% CI: 1.00–1.13; *p* = 0.059) and UTI (Adj.OR = 1.07; 95% CI: 0.99–1.15; *p* = 0.097). Pharmacy staff who believed that antibiotics were effective against the evaluated minor infections were more likely to supply antibiotics for acute sore throat (Adj. OR = 1.2; 95% CI: 1.08–1.34; *p* = 0.001), the common cold and cough (Adj.OR = 1.15; 95% CI: 1.03–1.28; *p* = 0.014), wound infections (Adj.OR = 1.15; 95% CI: 1.05–1.28; *p* = 0.004) and UTI (Adj.OR = 1.13; 95% CI: 0.99–1.27; *p* = 0.061). Pharmacy staff with any form of reported pharmacy qualification were less likely to supply antibiotics without a prescription for common infections, including acute sore throat (Adj. OR = 0.33; 95% CI: 0.13–0.80; *p* = 0.014), common cold and cough (Adj.OR = 0.31; 95% CI: 0.13–0.72; *p* = 0.007) and diarrhoea (Adj.OR = 0.36; 95% CI: 0.14–0.97; *p* = 0.043).

## 3. Discussion

These findings are part of the first national survey conducted among Sri Lankan community pharmacies aiming to evaluate pharmacy staff’s knowledge about antibiotics, and their attitudes towards antibiotics supply for common infections. The focus of the current study was to investigate attitudes towards antibiotic supply without prescriptions for common infections and to evaluate the association between attitudes and supply of antibiotics. This study included all different types of national retail community pharmacies, pharmacists with different professional qualifications, and staff responsible for managing pharmacies without any pharmacy qualification. The high response rate (78%) was also a strength of this study.

Most of the respondents did not believe that they were professionally competent to supply antibiotics without a prescription and monitor people’s antibiotic use. They did not believe that antibiotics were effective against different infections, including viral infections. Whilst they had positive beliefs about the effectiveness of antibiotics, they lacked beliefs about their own competency. This is an interesting finding that may highlight their limited knowledge about antibiotics and limited clinical experience, which may be a reflection of the qualifications held by the pharmacy staff, where only 2% held a bachelor’s degree in pharmacy, and the majority of the pharmacists had efficiency and proficiency training. 

The adjusted multiple logistic regression models showed that pharmacy staff’s beliefs about their professional competency to supply antibiotics without a prescription and monitor antibiotics use by people, and their beliefs about the effectiveness of antibiotics for minor infections were strongly associated with staff’s behaviour of supplying antibiotics without a prescription for such infections. One explanation for this observation could be that those respondents who believed in their own professional competency and the effectiveness of antibiotics were more likely to provide antibiotics without a prescription. This observation may be further supported by the finding that a pharmacist with any form of recognised pharmacy qualification in Sri Lanka was less likely to supply antibiotics without a prescription for possible viral infections. 

However, supplying antibiotics without a prescription is an illegal practice in the country and supplying antibiotics inappropriately for possible viral infections is worrying, because it highlights pharmacy staff’s limited knowledge about antibiotics and their appropriate use. Comparable to these observations, pharmacists’ and non-pharmacist staff’s poor knowledge has been reported elsewhere [28]. Barker et al. in 2017 have also reported that the factors associated with antibiotic supply without a prescription, especially in LMICs, are the extent of pharmacy education and the clinical training [21]. 

The poor clinical knowledge of pharmacy staff in this survey could possibly reflect the extent of their pharmacy training, because most respondents (53%) reported that they had completed apprentice pharmacy training with no tertiary education or clinical training [30]. Pharmacy staff’s beliefs associated with inappropriate antibiotics supply for possible viral infections was also supported by mystery shopper visit findings to the same pharmacies [18]. Similar findings have also been reported in South Korea, where pharmacists believed that antibiotics are helpful in treating the paediatric common cold [31]. 

Whilst our findings demonstrated that supply of antibiotics without a prescription from community pharmacies for common infections is widespread in Sri Lanka, these figures are lower when compared to our mystery shopper visits to the same pharmacies. The simulated client visits with direct antibiotic product requests and symptom-based antibiotic requests for four common infections (acute sore throat, common cold and cough, diarrhoea and uncomplicated UTI) revealed a higher prevalence of antibiotic supply, at 61% [19] and 41% [18], respectively, compared to the self-reported survey finding for the identical conditions and pharmacies (19.4%, 47/242). This difference is probably due to social desirability bias of self-reported surveys [32]. The detected discrepancies between self-reported and observed behaviour have also been demonstrated in a systematic review assessing performance of pharmacies and drug stores in LMICs in the Asian region [33]. For instance, in Vietnam, only 20% of the pharmacists reported they would supply antibiotics without a prescription for children with a viral URTI, but the observed supply was 83% in mystery shopper visits [34].

Availability of a pharmacist in the pharmacy significantly impacted antibiotic supply without a prescription. It was found that pharmacy staff with any formal pharmacy qualification recognised by the Sri Lankan government were less likely to supply antibiotics without a prescription for common infections, as they are more likely to be aware of the legal requirements of antibiotic supply. This finding was also supported by mystery shopper studies conducted in the same pharmacies [18,19], and in another study which showed that pharmacists with a knowledge of the legal aspect of antibiotic supply were less likely to dispense antibiotics without a prescription [28].

The findings of this study have identified a need for interventions to improve pharmacists’ and pharmacy staff’s knowledge related to appropriate antibiotic use and supply, and influence their beliefs about antibiotics and possibly behaviour (antibiotic supply). This will help to support appropriate antibiotic use in the community in the long run. 

An important limitation of this study was that the data were self-reported, and there was therefore the possibility for social desirability bias, and issues with recall. The proportion of pharmacists and non-pharmacist staff who reported supplying antibiotics without a prescription might be an underestimation of what is occurring in real practice. 

## 4. Materials and Methods

This was a cross-sectional national survey conducted among community pharmacy staff throughout Sri Lanka between December 2016 and September 2017. This study was part of a larger study, investigating knowledge, attitudes and dispensing practices regarding antibiotics among community pharmacy staff in Sri Lanka. 

A total of 369 community pharmacies throughout the country were selected for study participation. The sample size estimation has been described previously [18,19,28]. A systematic sampling of community pharmacies was carried out throughout the country, including all types of semi-government (Rajya Osusala) and private (single, chain and pharmacies in private hospitals) pharmacies. The study sample was achieved using a proportionate sampling technique in each province, as described below and elsewhere [18,19,28]. 

Pharmacies included in this survey were randomly selected from all nine provinces (sampling frame) using a simple random sampling technique. The sample of pharmacies which had legal permission to function in each province was derived from the list of pharmacies from the Pharmacy Directory maintained by the National Medicine Regulatory Authority (NMRA) (as of April 2016) [35]. A random point in the roadmap in each province was selected and the first pharmacy on that road was identified as the index pharmacy. Pharmacies were approached throughout the province until the provincial quota was filled. A similar approach was continued in all other provinces [18,19,28]. A validated, self-administered and structured questionnaire was used to collect data [28]. 

### 4.1. Measures

The study questionnaire was developed based on the results of previous studies conducted in other countries [24,32,36,37,38,39,40,41], which investigated community pharmacy staff’s knowledge, attitudes and practices regarding antibiotics. Questionnaire development, questionnaire validation (face and content validity), sampling strategy and data collection have been previously reported [28]. Face and content validity, including, language, organisation, appropriateness and logical sequence of items, accuracy, clinical terminologies, completeness and meaning of the items, and comprehension of the items in the questionnaire were assessed by a panel of experts from Australia and Sri Lanka, resulting in minor amendments to the final questionnaire [28]. The questionnaire was developed in English and translated into the two primary languages (Sinhala and Tamil) spoken in Sri Lanka using the forward-backward method [28].

Data on: (i) pharmacy staff’s personal and professional characteristics; (ii) their beliefs and attitudes towards antibiotic supply without a prescription; and (iii) supply of antibiotics without a prescription, for five common infections—acute sore throat, the common cold and cough, diarrhoea, wound infection and urinary tract infection (UTI), were collected using the self-administered questionnaire.

#### 4.1.1. Personal and Professional Characteristics 

These included age, gender, geographical location, level of pharmacy education, years of working experience in community pharmacy, employment type, employment status, type of pharmacy, and number of pharmacists and pharmacy assistants working at a given time in the pharmacy.

Community pharmacist was defined as a respondent with any of the following qualifications in pharmacy: graduate pharmacist (B.Pharm or BSc (Pharmacy)), proficiency certificate pharmacist, or diploma in pharmacy or efficiency certificate pharmacist (apprentice pharmacist). All groups of pharmacists have equal pharmacy practice rights in Sri Lanka. All other respondents who did not hold these qualifications were considered as non-pharmacist staff.

#### 4.1.2. Beliefs and Attitudes towards Antibiotic Supply without a Prescription 

Beliefs and attitudes were measured using 43 statements on a 5-point Likert scale with 1 = strongly disagree, 2 = disagree, 3 = neither agree nor disagree, 4 = agree and 5 = strongly agree. 

#### 4.1.3. Antibiotics Supply without a Prescription for Common Infections

The supply of antibiotics without a prescription within the week prior to survey completion by pharmacy staff for common infections (acute sore throat, common cold and cough, wound infections, UTI and diarrhoea) were assessed using a five-point Likert scale (1 = 0% or Never, 2 = 25% of prescriptions dispensed, 3 = 50% of prescriptions dispensed, 4 = 75% of prescriptions dispensed, 5 = 100% or Always; and an additional option “don’t know” was also included).

### 4.2. Data Analysis

Completed questionnaires were coded, reviewed for accuracy and entered into a database. Statistical Package for the Social Sciences (SPSS, Version 24.0, IBM Corp, Armonk, NY, USA) was used for all of the analyses. 

Descriptive analysis: Personal and professional characteristics, beliefs and attitudes towards antibiotic supply (including latent constructs) and antibiotics supply for common infections were presented either using mean ± SD or median interquartile range (IQR) for continuous data, or frequencies and percentages for nominal data. Factor analysis was performed to identify latent attitudinal factors influencing antibiotic supply without a prescription. One-way ANOVA was performed to evaluate for any difference in the attitudinal scores between the three different groups of respondents (proficiency pharmacists, efficiency pharmacists and non-pharmacists).

Factors influencing antibiotic supply for common infections were assessed using multivariate logistic regression using the backward elimination method provided in SPSS. The attitude constructs, and all the personal and professional characteristics were introduced together in the regression model. The regression outcome, antibiotic supply without a prescription for common infections was reported by adjusted β and the corresponding 95% confidence interval (CI). The level of significance was set as *p* < 0.05. The dependent and independent variables used in the regression models have been described below.

The pharmacist and non-pharmacist data were combined for the factor analysis and in the regression models because, as observed in this study (Table 1) and in earlier research [28], the non-pharmacist staff behave similarly to pharmacists and provide antibiotics without a prescription. Furthermore, there was a smaller number of non-pharmacist participants (*n* = 55) in the study.

#### 4.2.1. Dependent Variables (Antibiotic Supply without a Prescription for the Five Common Infections)

The 5-point Likert scale items assessing rate of antibiotic supply without a prescription in the week prior to the survey for acute sore throat, the common cold and cough, diarrhoea, wound infection and UTI were recoded into a dichotomous variable by treating responses from “25%” to “always” as antibiotic supplied (and given the value of “1”), and “never” as it was and given a value of “0”. The “don’t know” responses were left out of the regression analyses. All five recoded dichotomous variables representing the five conditions were summed to create an overall summative score for non-prescription antibiotic supply for common infections (score 0–5), where “0” meant that no antibiotics were supplied without a prescription, and “1–5” meant that antibiotics were supplied without a prescription for one to five common infections.

#### 4.2.2. Independent Variables

The association between pharmacy staff’s beliefs and attitudes related to antibiotic supply without a prescription and their professional characteristics with antibiotic supply for common infections was investigated. 

#### 4.2.3. Exploratory Factor Analysis

Exploratory factor analysis (EFA) was conducted to determine the dimensionality reduction of the variables measuring attitudes towards antibiotic supply without a prescription. In this study, there were 43 attitudinal items, and some were correlated with each other. EFA allows for the reduction of these items into meaningful factors and takes into account the correlation between the items. In EFA, relationships among sets of interrelated variables are examined and represented in terms of underlying factors. The construct validity was tested using the principal axis factor (PAF) method with oblimin rotation. Eigenvalue greater than 1 was used as a criterion to select the initial number of factors to be rotated. A cut-off value of 0.30 was set for factor loadings as inclusion criterion in a factor [29]. Varimax rotation factor analyses revealed a five-factor solution, which was consistent with the conceptual basis of the scale (Table 2). The reliability of each construct was computed using Cronbach’s alpha and average inter-item correlation. A reliability coefficient of ≥0.7 was considered acceptable [42]. The weighted factor-based scores were created taking into account the actual reported value on the Likert scale and factor loading value [29]. 

## 5. Conclusions

Whilst pharmacy staff are providing antibiotics to the public without a prescription, they appear to hold beliefs that antibiotics are ineffective against common infections, evaluated that they do not have the professional competency to supply and monitor the use of antibiotics provided without a prescription to people, and that supply of antibiotics should follow the legal requirements. However, they believe that the public should have an easy access to antibiotics.

Pharmacy staff’s beliefs about the effectiveness of antibiotics for common infections and their beliefs about their professional competency to supply antibiotics without a prescription and monitor antibiotic use by the public were associated with the supply of antibiotics without a prescription.

The findings highlight reasons that could contribute to inappropriate supply of antibiotics without a prescription and further emphasise an urgent need for educational and regulatory interventions to improve appropriate antibiotic dispensing practices in Sri Lanka. 

## Figures and Tables

**Table 1:** Socio-demographic and professional characteristics.

Characteristics	Frequency (%)			
	Overall	Pharmacists	Pharmacy	χ^2^ *p* Value
	*n* = 265	*n* = 210	Assistants *n* = 55	
Gender				0.931
Male	172 (64.9)	137 (65.2)	35 (63.6)	
Female	91 (34.3)	73 (34.8)	18 (32.7)	
Missing data	2 (0.8)	0	2 (3.6)	
Age groups (Years)				0.913
20–29	43 (16.2)	31 (14.8)	12 (21.8)	
30–39	103 (38.9)	85 (40.5)	18 (32.7)	
40–49	65 (24.5)	50 (23.8)	15 (27.3)	
≥50	50 (18.9)	43 (20.5)	7 (12.7)	
Missing data	4 (1.5)	1 (0.5)	3 (5.5)	
Geographical area				0.353
Urban	189 (71.3)	147 (70.0)	42 (76.4)	
Rural	76 (28.7)	63 (30.0)	13 (23.6)	
Level of Pharmacy Education				N/A
Proficiency ^a^	65 (24.5)	65 (31.0)	N/A	
Efficiency ^b^	140 (52.8)	140 (66.7)	N/A	
Degree ^c^	5 (1.9)	5 (2.4)	N/A	
Pharmacy trainee ^d^	11 (4.2)	N/A	11 (20.0)	
No pharmacy education	40 (15.0)	N/A	40 (71.7)	
Missing data	4 (1.5)	0	4 (7.3)	
Years of work experience in the community pharmacy				<0.001
≤1	21 (7.9)	8 (3.8)	13 (23.6)	
2–3	27 (10.2)	25 (11.9)	2 (3.6)	
4–5	34 (12.8)	30 (14.3)	4 (7.3)	
>5	178 (67.2)	144 (68.6)	34 (61.8)	
Missing data	5 (1.9)	3 (1.4)	2 (3.6)	
Employment type				0.332
Owner (pharmacist or non-pharmacists)	101 (38.1)	84 (40.0)	17 (30.9)	
Employee	161 (60.8)	126 (60.0)	35 (63.6)	
Missing data	3 (1.1)	0	3 (5.5)	
Employment status				0.823
Full time	219 (82.6)	175 (83.3)	44 (80.0)	
Part time	43 (16.2)	35 (16.7)	8 (14.5)	
Missing data	3 (1.1)	0	3 (5.5)	
Type of pharmacy				0.214
Rajya Osusala (Semi Government)	20 (7.5)	18 (8.6)	2 (3.6)	
Private chain pharmacy	116 (43.8)	88 (41.9)	28 (50.9)	
Single private pharmacy	118 (44.5)	97 (46.2)	21 (38.2)	
Pharmacies in Private hospitals	11 (4.2)	7 (3.3)	4 (7.3)	
Total number of registered pharmacists working in the pharmacy				0.651
None	7 (2.6)	5 (2.4)	2 (3.6)	
1	179 (67.5)	143 (68.1)	36 (65.5)	
≥2	72 (27.3)	59 (27.3)	13 (23.6)	
Missing data	7 (2.6)	3 (1.4)	4 (7.3)	
Number of pharmacists at any given time				0.53
0	7 (2.6)	4 (1.9)	3 (5.5)	
1	195 (73.6)	158 (75.2)	37 (67.3)	
2	23 (8.7)	18 (8.6)	5 (9.1)	
>2	15 (5.8)	15 (7.3)	0	
Missing data	25 (9.4)	15 (7.1)	10 (18.2)	

N/A—Not applicable. ^a^ Pharmacists with two years certificate or diploma qualification including 6 months hospital training. ^b^ Pharmacists with an apprentice training program under a trained pharmacist’s supervision. ^c^ Pharmacists with B.Pharm or BSc pharmacy qualification. ^d^ Individual registered for apprentice pharmacy program and undergoing in the training program.

**Table 2:** Exploratory factor analysis.

Factors	*n*	Mean (SD)	Median (IQR)	Factor Loading				
				Factor 1	Factor 2	Factor 3	Factor 4	Factor 5
Factor 1 Belief in professional competency to supply and monitor Dispensing antibiotics without prescription is not a problem in my pharmacy because,								
I have the ability to monitor appropriate use of antibiotics by patients	261	1.8 (0.83)	2 (1, 2)	0.892				
I have the ability to assess the patient’s need for antibiotics	261	1.8 (0.79)	2 (1, 2)	0.877				
I am well trained and have sufficient experience in dispensing antibiotics	262	1.8 (0.8)	2 (1, 2)	0.872				
I have the ability to monitor patients’ adherence to their antibiotic course	261	1.8 (0.82)	2 (1, 2)	0.839				
I have the ability to monitor patients for adverse drug reactions to antibiotics	261	1.8 (0.76)	2 (1, 2)	0.799				
I believe that antibiotics are safe medicines	262	1.9 (0.86)	2 (1, 2)	0.791				
I can properly consult patients on appropriate use of antibiotics	261	2.1 (1.05)	2 (1, 2)	0.759				
I know to prescribe antibiotics to the patients or refer them to doctor	261	1.9 (0.98)	2 (1, 2)	0.739				
I have a good knowledge of antibiotic therapy	262	1.8 (0.8)	2 (1, 2)	0.697				
I believe that antibiotics have no or few side effects	263	1.8 (0.83)	2 (1, 2)	0.680				
Factor 2 Belief in shared responsibility Promoting appropriate use of antibiotics is a shared responsibility of:								
Policy makers	256	4.2 (0.95)	4 (4, 5)		0.810			
Patients	256	3.9 (1.18)	4 (4, 5)		0.770			
Pharmacists	259	4.4 (0.77)	5 (4, 5)		0.699			
Doctors	259	4.5 (0.79)	5 (4, 5)		0.520			
Factor 3: Beliefs in effectiveness of antibiotics I believe antibiotics can speedup recovery of the following conditions, therefore I can dispense antibiotics without a prescription for:								
Wound infections	259	1.8 (0.98)	2 (1, 2)			0.843		
Common cold and cough	260	4.3 (0.85)	2 (1, 2)			0.834		
Diarrhoea	259	4.3 (0.85)	2 (1, 2)			0.824		
Acute sore throat	259	4.3 (0.82)	2 (2, 2)			0.807		
Fever	262	4.5 (0.66)	1 (1, 2)			0.781		
UTI	259	1.6 (0.76)	1 (1, 2)			0.731		
Factor 4 Access and availability								
Antibiotic should be dispensed without a prescription if I know that patient does not have enough time to see a doctor	262	1.7 (0.80)	2 (1, 2)				0.770	
Patients have right to obtain antibiotics from a pharmacy without a prescription, if they can’t see a doctor	263	1.9 (0.97)	2 (1, 2)				0.702	
Antibiotic should be dispensed without a prescription if I know that patient does not have enough money to see a doctor	262	1.8 (0.82)	2 (1, 2)				0.546	
I believe antibiotic can cure all bacterial infections, therefore I can dispense antibiotics for bacterial infections without a prescription	263	1.6 (0.81)	2 (2, 2)				0.475	
I should dispense antibiotics without a prescription, because in my opinion, antibiotic use has nothing to do with antibiotic resistance	262	1.5 (0.7)	1 (1,2)				0.446	
Antimicrobial education taught during pharmacy training program is sufficient to allow me to dispense antibiotics without a prescription	261	2.2 (1.1)	2 (1,3)				0.353	
Pharmacists should be encouraged to dispense antibiotics without a prescription in order to meet patients’ demand.	262	1.7 (0.98)	1 (1, 2)				0.312	
Factor 5 Attitudes towards appropriate and legal supply and use								
Dispensing antibiotics without a prescription should be more closely controlled by the authorities	261	4.1 (1.02)	4 (4, 5)					0.628
Patient should stop misusing antibiotics to minimise the occurrence of antibiotic resistance	262	4.4 (0.91)	5 (4, 5)					0.611
Patients should stop demanding antibiotics without a prescription from pharmacies	262	4.2 (0.95)	4 (4, 5)					0.465
I believe that as a pharmacist, I have the right to contribute towards the appropriate use of antibiotic in Sri Lanka	260	4.4 (0.89)	5 (4, 5)					0.444
Appropriate use of antibiotics would help to decrease resistance; therefore, I should not dispense antibiotics without a prescription.	262	4.2 (1.1)	4 (4,5)					0.371
Reliability Cronbach’s alpha				0.951	0.827	0.920	0.763	0.701

Note: Only the absolute values > 0.30 are reported in the table. Four items were excluded, because they were not loaded in the model (S1, S10, S18, S25). Seven items were excluded due to poor factor loading was (<0.3). All Factors had scale range between 1 = ‘strongly disagree’ and 5 = ‘strongly agree’. Factor 1, Professional competency to supply and monitor: pharmacy staff’s beliefs about their professional competency (e.g., due to their pharmacy training, knowledge, patient counselling ability and experiences) to supply antibiotics without a prescription and monitor people’s use of antibiotics (e.g., monitor side effects) without a prescription. Factor 2, Shared responsibility: staff’s beliefs that promoting appropriate use of antibiotics is a shared responsibility of various stakeholders including, policy makers, patients, pharmacists and doctors. Factor 3, Beliefs about effectiveness of antibiotics: beliefs about the effectiveness of antibiotics against conditions, such as wound infections, common cold and cough, diarrhoea, acute sore throat, fever and UTI. Factor 4, Access and availability: Pharmacy staff’s opinions about the ability of people to easily access and receive antibiotics without a prescription from pharmacies to meet their needs if they do not have enough money or time to see a doctor. And beliefs that antibiotics cure all bacterial infections, ABR is not a problem, and they were sufficiently trained on antibiotics in their pharmacy training. Factor 5, Appropriate and legal supply and use: participants’ attitudes towards antibiotics supply control strategies and included beliefs that antibiotic supply without a prescription should be controlled by authorities, and that patients’ demands and misuse of antibiotics and pharmacists’ contribution towards appropriate use of antibiotics should be stopped.

**Table 3:** Weighted factor-based scale statistics.

Factors	Range of the Weighted Scale	Mid-Point for the Weighted Scale	Mean (SD)	Median (IQR)	Kurtosis Value	Skewness
Factor 1: Professional competency to supply and monitor	7.9–31.8	19.8	14.7 (5.7)	15.9 (8.6, 16.6)	0.62	0.77
Factor 2: Shared responsibility	2.8–14.0	8.4	11.8 (2.1)	11.7 (11.2, 14.0)	2.34	−1.22
Factor 3: Beliefs in effectiveness of antibiotics	4.8–24.1	14.4	8.0 (3.4)	8.1 (4.8, 9.6)	1.98	1.16
Factor 4: Access and availability	2.5–12.6	7.5	10.7 (1.7)	11.0 (10.1, 12.0)	4.08	−1.58
Factor 5: Appropriate and legal supply and use	2.5–12.6	7.5	10.7 (1.7)	11.0 (10.1, 12.0)	4.08	−1.58

**Table 4:** Factors related to antibiotic supply for different infections.

	Conditions for Which Antibiotics Supplied without a Prescription Last Week. Adj.OR (95% CI)					
Predictors	Acute Sore Throat (*n* = 218)	Common Cold and Cough (*n* = 220)	Wound Infection (*n* = 221)	UTI (*n* = 220)	Diarrhoea (*n* = 220)	Overall Supply (*n* = 221)
Antibiotic supply without prescription in the last week (*n* = 265)						
Never dispensed	217 (81.9) ^a^	214 (80.8) ^a^	199 (75.1) ^a^	231 (87.2) ^a^	227 (85.7) ^a^	179 (67.5) ^a^
Dispensed	36 (13.6) ^a^	42 (15.8) ^a^	56 (21.1) ^a^	23 (8.7) ^a^	27 (10.2) ^a^	80 (30.2) ^a^
Missing data	12 (4.6) ^a^	9 (3.4) ^a^	10 (3.8) ^a^	11 (4.2) ^a^	11 (4.2) ^a^	6 (2.3) ^a^
Factor 1: Professional competency to supply and monitor		1.08(1.01–1.15) *	1.06 (1.00–1.13) ^NS^	1.07 (0.99–1.15) ^NS^		1.12 (1.05–1.19) **
Factor 2: Shared responsibility					0.80 (0.67–0.96) *	
Factor 3: Beliefs about effectiveness of antibiotics	1.2 (1.08–1.34) **	1.15 (1.03–1.28) *	1.15 (1.05–1.28) **	1.13 (0.99–1.27) ^NS^	-	1.11 (1.01–1.22) *
Pharmacy education						
Non-Pharmacists	1	1			1	1
Pharmacist	0.33 (0.13–0.80) *	0.31 (0.13–0.72) **			0.36 (0.14–0.97) *	0.48 (0.21–1.08) ^NS^
Age	-	-			-	0.95 (0.92–0.98) **

^a^ Frequency (%). ^NS^ Not significant; Significant at * <0.05; ** <0.01. Other predictors adjusted in the model: Knowledge about: effectiveness of antibiotics for minor infections, what is an antibiotic, adverse effects of antibiotics and ABR. Attitudes: Supply or access, Supply control. Socio-demographic characteristics: gender, geographical area, employment type, employment status, type of pharmacy, number of pharmacy assistants work at a time, number of pharmacists work at a time and years of work experience in the community pharmacy setting.

## Data Availability

All relevant data are included in the manuscript.

## Supplementary Materials

The following are available online at https://www.mdpi.com/article/10.3390/antibiotics10060647/s1, Table S1: Attitudinal factor scores between the three different groups of pharmacy staff.
