# Supplementary material for: Factors Related to Antibiotic Supply without a Prescription for Common Infections: A Cross-Sectional National Survey in Sri Lanka
Source: Antibiotics (Basel). 2021 May 28;10(6):647. doi: 10.3390/antibiotics10060647 (PMC8227992; doi:10.3390/antibiotics10060647)
Supplement: Supplementary file 1 [file antibiotics-10-00647-s001.zip › antibiotics-1183670-supplementary.pdf]

antibiotics

MDPI

Article

Factors Related to Antibiotic Supply Without a Prescription for Common Infections: a Cross-Sectional National Survey in Sri Lanka

Shukry Zawahir [1,3,*], Sarath Lekamwasam [2] and Parisa Aslani [3]

[1] Central Clinical School, Faculty of Medicine and Health, The University of Sydney, Sydney, NSW 2006 Australia
[2] Population Health Research Centre, Department of Medicine, Faculty of Medicine, University of Ruhuna, Galle 80000, Sri Lanka; slekamwasam@gmail.com
[3] The University of Sydney School of Pharmacy, Sydney, NSW 2006, Australia; parisa.aslani@sydney.edu.au
* Correspondence: shukry2010@gmail.com; Tel.: +61 290363110

Table S1. Attitudinal factor scores between the three different groups of pharmacy staff

Factors	Non-Pharmacist Staff Mean (SD)	Pharmacists (Efficiency) Mean (SD)	Pharmacists (Profficiency) Mean (SD)	F statistics	*P* value
Factor 1: Professional competency to supply and monitor	14.1 (5.8)	15.0 (5.9)	14.6 (5.4)	0.43	0.648
Factor 2: Shared responsibility	11.6 (2.3)	11.8 (2.1)	12.0 (2.0)	0.57	0.569
Factor 3: Beliefs in effectiveness of antibiotics	7.7 (3.1)	8.1 (3.5)	8.1 (3.3)	0.27	0.762
Factor 4: Access and availability	10.7 (1.5)	10.8 (1.7)	10.7 (1.7)	0.05	0.952
Factor 5: Appropriate and legal supply and use	10.7 (1.5)	10.8 (1.7)	10.7 (1.7)	0.05	0.952

Proficiency: Pharmacists with two years certificate or diploma qualification including 6 months hospital training, or Pharmacists with B.Pharm or BSc pharmacy qualification. Efficiency: Pharmacists with an apprentice training program under a trained pharmacist's supervision.
